# Prevalence of Respiratory Viral Infections in Deceased Persons during the COVID-19 Pandemic Season 2021–2022: A Population-Based Observational Study

**DOI:** 10.3390/v16040533

**Published:** 2024-03-29

**Authors:** Camino Trobajo-Sanmartín, Ana Navascués, Miguel Fernández-Huerta, Iván Martínez-Baz, Itziar Casado, Carmen Ezpeleta, Jesús Castilla

**Affiliations:** 1 Instituto de Salud Pública de Navarra, 31003 Pamplona, Spainitziar.casado.buesa@navarra.es (I.C.); 2 CIBER Epidemiología y Salud Pública (CIBERESP), Instituto de Salud Carlos III, 28029 Madrid, Spain; 3 Instituto de Investigación Sanitaria de Navarra (IdiSNA), 31008 Pamplona, Spain; ana.navascues.ortega@navarra.es (A.N.); miguel.fernandez.huerta@navarra.es (M.F.-H.); cezpeleb@navarra.es (C.E.); 4 Clinical Microbiology Department, Hospital Universitario de Navarra, 31008 Pamplona, Spain

**Keywords:** respiratory virus, respiratory viral infection, mortality, influenza, COVID-19, respiratory syncytial virus, rhinovirus, metapneumovirus

## Abstract

Although the omicron variant of SARS-CoV-2 circulated intensely during the 2021–2022 season, many patients with severe acute respiratory disease tested negative for COVID-19. The aim of this study was to assess the presence of different respiratory viruses in deceased persons. The proportion of deceased persons with respiratory viral infections in the 2021–2022 season in Navarre, Spain, was estimated considering all deaths caused by confirmed COVID-19 according to the epidemiological surveillance and the results of multiplex PCR tests for respiratory viruses performed in a sample of deceased persons with a cause of death other than COVID-19. Of 3578 deaths, 324 (9.1%) were initially reported as caused by pre-mortem confirmed COVID-19. A sample of 242 persons who died by causes other than COVID-19 were tested post-mortem; 64 (26.4%) of them were positive for any respiratory virus: 11.2% for SARS-CoV-2, 5.8% for rhinovirus, 3.7% for human coronavirus, 2.5% for metapneumovirus, 1.7% for respiratory syncytial virus, 1.7% for parainfluenza, 1.2% for influenza, and less than 1% each for adenovirus and bocavirus. Combining both approaches, we estimated that 34.4% of all deceased persons during the study period had a respiratory viral infection and 19.2% had SARS-CoV-2. Only 33.3% (9/27) of SARS-CoV-2 and 5.0% (2/40) of other viruses detected post-mortem had previously been confirmed pre-mortem. In a period with very intense circulation of SARS-CoV-2 during the pandemic, other respiratory viruses were also frequently present in deceased persons. Some SARS-CoV-2 infections and most other viral infections were not diagnosed pre-mortem. Several respiratory viruses may contribute to excess mortality in winter.

## 1. Introduction

Mortality increases are common during the respiratory viral infection seasons. Annual epidemics of influenza and respiratory syncytial virus (RSV) have been related to excess mortality [1,2,3]. Other respiratory viruses have also been related to deaths, but their role in excess mortality has not been well established [4,5,6].

Between 2020 and 2022, severe acute respiratory syndrome coronavirus 2 (SARS-CoV-2) was the dominant respiratory virus worldwide, displacing and changing the epidemiological and clinical features of other respiratory viruses [7,8]. During the 2021–2022 season, the omicron variant of SARS-CoV-2 circulated intensively, infecting a large proportion of the population in Europe and other regions [9,10,11,12,13]. Nevertheless, many patients with severe acute respiratory disease tested negative for SARS-CoV-2, and other respiratory viruses were not routinely ruled out. Epidemiological surveillance in Europe showed that the influenza activity increased in 2021–2022 compared to the 2020–2021 season, but it was still clearly lower than the influenza activity observed in seasons before the COVID-19 pandemic [12].

The impact of the SARS-CoV-2 omicron variant epidemic on mortality was modulated by high COVID-19 vaccination coverage with very effective vaccines to prevent death among COVID-19 cases [14]. Furthermore, the severity of COVID-19 cases due to the omicron variant was found lower than that due to the previous circulating SARS-CoV-2 variants [15].

In Navarre, Spain, the 2021–2022 season was characterized by a very high incidence of COVID-19 confirmed cases; 27% of the population was confirmed in the healthcare system [11], and a very low incidence of laboratory-confirmed influenza and related mortality was observed, despite the increased sensitivity of detection [16,17]. Full COVID-19 vaccination coverage reached 83.9%, and 51.6% had one booster dose at the beginning of the season [11]. Influenza vaccination coverage reached 78% among people aged 65 years and older [16].

Since only a small percentage of deceased people were usually tested for respiratory viruses before they died, the impact of respiratory viruses on mortality is not well known [1,18]. Furthermore, mortality statistics by cause of death largely underestimate the role of respiratory viruses in mortality [18]. While a unique source of information did not provide a complete view of the respiratory-virus-related mortality, a combination of two independent sources may provide a more complete view.

The objective of this study was to assess the presence of the different respiratory viruses in deceased persons during the 2021–2022 season.

## 2. Materials and Methods

### 2.1. Study Design

A population-based observational study analyzed the prevalence of respiratory virus infection in deceased persons in the Navarre region (~660,000 inhabitants), Spain, in the 2021–2022 season. This study combined two complementary sources of information: (1) laboratory-confirmed cases of respiratory viral infections obtained from the enhanced epidemiological and virological surveillance, and (2) post-mortem recruitment and testing of a sample of persons who died of natural causes.

### 2.2. Epidemiological and Virological Surveillance of Resporatory-Viral-Infection-Related Deaths

In this study, enhanced epidemiological and virological surveillance was used to identify all patients with a confirmed respiratory virus infection between October 2021 and September 2022, who died within 30 days of diagnosis. During this period, a diagnostic test was available free of charge for all hospitalized patients and seriously ill patients with respiratory symptoms who consulted primary healthcare centers. Nasopharyngeal samples were tested by quantitative reverse-transcription polymerase-chain-reaction (RT-qPCR) for COVID-19, influenza, and respiratory syncytial virus (RSV). Multiplex RT-qPCR for other respiratory viruses was performed according to the medical criteria in some hospitalized patients. The enhanced epidemiological surveillance included the electronic reporting of all clinical diagnoses of acute respiratory illness, laboratory test results, and data on hospital admissions and mortality of cases. As part of the epidemiological surveillance, medical doctors reviewed all hospital admissions and deaths of patients with confirmed SARS-CoV-2 infection during the three months after the positive test. Deaths of confirmed COVID-19 cases that occurred outside the hospital were also considered. Deaths due to COVID-19 were considered to be those that occurred in patients with confirmed SARS-CoV-2 infection in whom the infection was a direct cause of death [19].

### 2.3. Post-Mortem Recruitment and Testing

Trained professionals of the most established funeral company in the region recruited a sample of persons who died of natural causes from December 2021 to 15 June 2022. Since the manipulation of the corpses of persons who died from COVID-19 was not permitted in Spain during the study period, individuals who were reported as having died from COVID-19 were not included in the post-mortem study. Therefore, the criteria for inclusion in the post-mortem study were: persons of all ages who had died of natural causes other than COVID-19; the closest relative signed the written informed consent, the body was prepared at the participating funeral company facilities, and the circumstances of the funeral preparations allowed the professionals to carry out the recruitment and swabbing.

Double swab specimens, nasopharyngeal and pharyngeal, were obtained from each individual before the body was prepared for burial, and both samples were introduced in the same viral transport media. The specimens were stored at 4 °C until processing. All swabs were tested in the Clinical Microbiology Department of the University Hospital of Navarre using multiplex RT-qPCR (AllplexTM respiratory panels, Seegene, Seoul, South Korea). These panels detected influenza A and B, RSV, SARS-CoV-2, adenovirus, enterovirus, metapneumovirus, parainfluenza virus 1–4, human bocavirus, rhinovirus, and human coronavirus (HCoV) 229E, NL63 and OC43. All influenza-positive and SARS-CoV-2-positive samples with a cycle threshold less than or equal to 30 were sequenced for genetic characterization. Sequencing results were deposited in the Global Initiative on Sharing All Influenza Data (GISAID) database.

### 2.4. Other Sources of Information and Variables

Demographic information, hospital admissions, and laboratory-confirmed diagnoses of respiratory viruses within 30 days before death were retrieved from electronic healthcare databases. The presence of respiratory symptoms before dying was obtained from clinical records of prior hospitalizations or consultations and was completed with information reported by relatives. Influenza and COVID-19 vaccination status was obtained from the regional vaccination register. All information referred to the same patient was linked using the individual identification number.

### 2.5. Statistical Analysis

The proportion of deaths with non-SARS-CoV-2 respiratory viral infection in the period from 1 December 2021 to 15 June 2022 was estimated using the percentages of positive results in post-mortem tests. The proportion of SARS-CoV-2-positive individuals among the deceased persons in the population was estimated by adding to the percentage of deaths from COVID-19 in the population during the study period and the corresponding proportion of SARS-CoV-2-positive results in the post-mortem tests for deceased persons who did not meet the criteria of death from COVID-19. The proportion of any respiratory virus in deceased persons in the population was corrected using the revised proportion of SARS-CoV-2.

The association between categorical variables was assessed using the Chi-squared test or the Fisher exact test when appropriate. Statistical significance was defined at *p* < 0.05.

## 3. Results

### 3.1. Description of the Study Population

**Figure 1:**
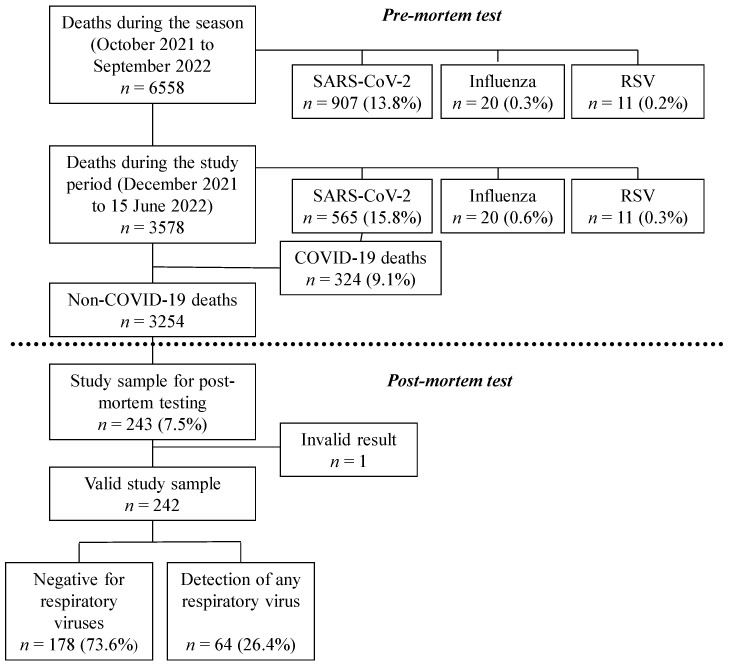
Study flow chart.

From October 2021 to September 2022, 6558 deaths were recorded in Navarre, of which 907 (13.8%) had a previous positive test for SARS-CoV-2, 20 (0.3%) for influenza and 11 (0.2%) for RSV within 30 days before death. In the study period, from December 2021 to 15 June 2022, 3578 deaths were registered, 565 (15.8%) had a previous positive result for SARS-CoV-2 within the 30 days before death, and 324 (9.1%) deaths were considered due to COVID-19; therefore, they were excluded from the post-mortem testing activity. Post-mortem specimens were obtained from 243 (7.5%) deceased persons and were processed by RT-qPCR, providing valid results in 242 of them (Figure 1).

The mean age of the deceased persons with valid post-mortem result was 81 years (median 85 years, range 42 to 108 years); 120 (49.6%) were women, 109 (45.0%) presented respiratory symptoms, and 127 (52.5%) had been hospitalized within 30 days before death.

### 3.2. Prevalence of Respiratory Virus Infection from Post-Mortem Tests

A positive RT-qPCR result for one or more respiratory viruses was obtained from 64 (26.4%) deceased persons who were tested post-mortem: 27 (11.2%) had a positive result for SARS-CoV-2, 14 (5.8%) for rhinovirus, 9 (3.7%) for HCoV (7 HCoV-OC43 and 2 HCoV-229E), 6 (2.5%) for metapneumovirus, 4 (1.7%) for RSV (1 subgroup A and 3 subgroup B), 4 (1.7%) for parainfluenza virus (3 parainfluenza-4 and one parainfluenza-2), 3 (1.2%) for influenza A(H3N2), 2 (0.8%) for adenovirus, and 1 (0.4%) for bocavirus (Table 1).

### 3.3. Influenza and SARS-CoV-2 Characterisation

Two of the three influenza A(H3N2) strains could be characterized and both were A/Bangladesh/4005/2020(H3N2)-like. Of the 27 SARS-CoV-2 confirmed infections, 10 (37.0%) were sequenced with the following results: three B.1.17, three BA.2, and one each of BA.1.18, BA.2.12.1, BA.5.1, and AY.43.

### 3.4. Co-Infections with More Than One Respiratory Virus

Co-infection by two different viruses was identified in 9.4% (6/64) of cases. Three cases presented co-infection by SARS-CoV-2 and another respiratory virus (parainfluenza-2, parainfluenza-4 or rhinovirus), 3 co-infections were one each positive for influenza A(H3N2) and rhinovirus, rhinovirus and parainfluenza-4, and metapneumovirus and HCoV-OC43. The frequency of co-infections was not significantly different from the expected according to the frequency of each specific virus.

### 3.5. Prevalence Estimates of Respiratory Viral Infections in Deceased Persons of the Population

SARS-CoV-2 was detected in 42.2% of the deceased persons who had any respiratory virus in the post-mortem swabs. Given that COVID-19 was the cause of 9.1% of all deaths registered during the study period and these deaths were excluded from the post-mortem testing, we estimated that 34.4% of the people who died had a respiratory virus infection, 19.2% had SARS-CoV-2 infection, and 16.5% had a virus other than SARS-CoV-2 (Table 1).

### 3.6. Characteristics of Deceased Persons with a Respitatory Viral Infection

The frequency of post-mortem detection of any respiratory virus did not differ by sex, reported respiratory symptoms, hospitalization within 30 days before death, or influenza vaccination status. The proportion of deceased persons with post-mortem detection of any respiratory virus increased significantly with age (*p* = 0.048), reaching 37.3% in those aged 90 years and over.

SARS-CoV-2 and other respiratory viruses were detected throughout the study period, with peaks in the percentage of positive results for SARS-CoV-2 in February and April, and for viruses other than SARS-CoV-2 in December, April, and May. Among deceased persons confirmed for any virus other than SARS-CoV-2, 60% (24/40) had reported respiratory symptoms.

Most of the post-mortem testers had received any dose of COVID-19 vaccine (95.9%), 81.0% had received a booster dose after full vaccination, and 71.1% had received the 2021–2022 influenza vaccine (71.1%). More doses of COVID-19 vaccination were associated with a lower probability of detection of a virus other than SARS-CoV-2 (*p* = 0.022) but not of SARS-CoV-2 (*p* = 0.523) (Table 2). Influenza vaccination was not associated with different probability of post-mortem detection of respiratory viruses. The three influenza infections detected post-mortem were vaccinated for influenza (*p* = 0.714).

### 3.7. Comparison of the Results of Clinical and Post-Mortem Detections of Respiratory Viral Infections

Of the 64 deceased persons with positive post-mortem results, 11 (17.2%) had received this diagnosis before death, 33.3% (9/27) of those due to SARS-CoV-2 and 5.0% (2/40) of those due to other viruses. However, including deaths from COVID-19 that were not tested post-mortem in the calculation, the corrected percentage of SARS-CoV-2 infections detected pre-mortem increased to 63.3%.

Nine of the 13 (69.2%) persons with a pre-mortem diagnosis of SARS-CoV-2 within 30 days before death were confirmed positive in the post-mortem test. The two patients confirmed pre-mortem with RSV had a similar positive result in the post-mortem test, but one person with an influenza positive result 13 days before death was influenza-negative in the post-mortem test. On the other hand, one patient with a negative result during a hospital admission presented a positive result for influenza virus in the post-mortem test five days later.

## 4. Discussion

During the 2021–2022 season, a period with especially intense circulation of SARS-CoV-2, isolates of SARS-CoV-2 and other respiratory viruses were detected in an appreciable proportion of deceased persons. We estimate that 34.4% of all deceased persons had any respiratory viral infection, 19.2% had SARS-CoV-2, and 16.5% had another virus. Despite the high availability of diagnostic tests for SARS-CoV-2 and the high proportion of the population confirmed for COVID-19 in the healthcare system (27%) [11], we estimate that about one in three SARS-CoV-2 infections in people who die was not detected pre-mortem, suggesting a higher presence of SARS-CoV-2 than indicated by the clinical diagnoses. Underdetection of cases was more pronounced for other respiratory viruses, since only 5% of infections detected post-mortem had been confirmed pre-mortem. A similar percentage (7%) was observed in a pre-pandemic season [18].

This study provides comparable estimates of the prevalence of several respiratory viruses regardless of the clinical practice. The results highlight that while SARS-CoV-2 was highly suspected and most cases were detected, most of the other respiratory viruses remained undiagnosed throughout the hospital admission process. Therefore, clinical diagnoses of non-SARS-CoV-2 respiratory viruses underestimate their real incidence as compared to the incidence of diagnosed SARS-CoV-2 infections.

Non-pharmacological preventive measures that had been applied during the COVID-19 pandemic were relaxed in the 2021–2022 season, resulting in an intense circulation of SARS-CoV-2 [20,21], and this virus displaced other respiratory viruses like influenza or RSV, which circulated scarcely [7,8,16,22]. Similarly, SARS-CoV-2 was the more frequent virus detected among deceased persons. However, our results showed that several respiratory viruses other than SARS-CoV-2 were also commonly found. These other viruses may be more frequently found in other seasons without dominance of SARS-CoV-2 [18].

Rhinovirus was the second most frequent virus detected (5.8%), which is usually found in the population as a cause of mild illness, as it was found in other studies [23]. However, rhinovirus has also been associated with death and longer hospitalization in the elderly, although, the role of this virus as a cause of death remains unclear in many cases [24,25,26,27].

The proportion of deceased persons with a positive result for respiratory virus increased with age, probably due to the immunosenescence that reduces the immune response to infections. In older people, the risk of severe diseases caused by viral infection increases and the viral clearance slows down [28]. Other authors have suggested the role of viral persistence as potential cause of death [29].

The paradoxical association between COVID-19 vaccination and a lower probability of virus detection other than SARS-CoV-2 may be explained because deaths caused by COVID-19 were excluded from the post-mortem testing, vaccinated people took more care to avoid exposure to respiratory viruses, and the possible nonspecific effect of the vaccines [30].

The three cases confirmed for influenza, all for influenza A(H3N2), had been vaccinated against influenza, although this association was not statistically significant. This finding is consistent with the genetic differences described between the A/Bangladesh/4005/2020(H3N2)-like circulating strain and the A/Cambodia/e0826360/2020(H3N2)-like vaccine component as well as the low influenza vaccine effectiveness found in this season against the influenza A(H3N2) subtype [17,31,32].

Most respiratory viral infections detected in deceased persons had not been diagnosed pre-mortem, since about half of them had not been hospitalized. Even among those who died in hospital, many were not tested for respiratory virus. The lack of testing means the role of respiratory viral infection in mortality was underestimated. Klein et al. analyzed 1231 persons who died during the first phase of the COVID-19 pandemic and were unsuspicious for SARS-CoV-2 infection, and found 29 positive individuals in the post-mortem test [33]. During an influenza epidemic in Spain, 47% of the deceased patients tested positive for respiratory virus infection post-mortem, but only 7% of them had been diagnosed pre-mortem [18]. These results suggest that respiratory viruses may be underreported as a cause of death.

The proportion of deceased persons with a positive result for respiratory virus was consistent with the excess mortality observed in the study region during the 2021–2022 season compared to the rest of the year and previous years [11,34], which supports some contribution of these infections to mortality.

Co-infections by more than one respiratory virus were detected in 9.4% of post-mortem positive cases with SARS-CoV-2, and rhinovirus was the most frequently detected virus in these cases. This result was consistent with other studies that found co-infection of different respiratory viruses in the population, since all these viruses share the same transmission mechanism. Vulnerability to different respiratory viral infections may be increased in people near to death, and some co-infections may be associated with increased disease severity [13,25,35,36,37,38,39].

The main strength of this study is the population-based approach that combined two information sources: (1) the enhanced epidemiological and virological surveillance, including deaths related to viral infections, and (2) the analysis of a sample of deceased persons regardless of whether they had a medical diagnosis before dying. Additionally, these results were compared to pre-mortem diagnoses of respiratory viral infections. Our study provides a more complete view than studies evaluating mortality among people with a confirmed diagnosis of respiratory viral infection, because these studies overlook deaths in people without medical care before dying [3,6,24], and than studies based on coronial post-mortem examinations that are less representative of all deaths [37,38,39,40,41,42].

The findings of the present study highlight the necessity of more studies on the impact of respiratory viruses on mortality and implementing integrate surveillance and prevention strategies for respiratory viral infections [43].

This study has some limitations. Viral detection in a deceased person does not necessarily mean that infection was the cause of death, because respiratory viral shedding is possible in asymptomatic people [44]. However, the presence of a pathogenic virus in a person who died is suggestive of the role of this virus as a contributing factor or trigger of death. The ability to eliminate viral colonization may also be lost as an indicator of an insufficient immunological response frequently associated with the proximity of the end of life. The study included a non-random sample of 7.5% of all deaths not reported as COVID-19-related during the study period in the region; therefore, selection bias is possible. False-negative results are possible in post-mortem tests since testing conditions of specimens obtained post-mortem may not be optimal. People who died from COVID-19 were excluded from the post-mortem study, but this bias was corrected in the estimates of the proportion of SARS-CoV-2-positive deceased persons.

## 5. Conclusions

In conclusion, despite the predominance of SARS-CoV-2 during the 2021–2022 season, the significant presence of other respiratory viruses in deceased persons was also found. Despite the large number of diagnostic tests performed, some of the SARS-CoV-2 infections and most of the other respiratory viral infections detected post-mortem had not been diagnosed pre-mortem, so it could be thought that the role of respiratory viruses in mortality may be underestimated. In addition to SARS-CoV-2 and influenza, other respiratory viruses may contribute to excess mortality in winter.

## Figures and Tables

**Table 1:** Results of the multiplex quantitative reverse-transcription polymerase chain reaction test performed in deceased persons.

	Cases among Deceased Persons Tested, *n* (*n* = 242)	Proportion of All Positive Samples % (*n* = 64)	Cases among Deceased Persons Tested, *%* (*n* = 242)	Estimated Proportion of All Deaths in the Population, *%*
SARS-CoV-2	27	42.2	11.2	19.2
Rhinovirus	14	21.9	5.8	5.8
Coronavirus 229E or OC43	9	14.1	3.7	3.7
Metapneumovirus	6	9.4	2.5	2.5
Respiratory syncytial virus	4	6.3	1.7	1.7
Parainfluenza virus	4	6.3	1.7	1.7
Influenza	3	4.7	1.2	1.2
Adenovirus	2	3.1	0.8	0.8
Bocavirus	1	1.6	0.4	0.4
All non-SARS-CoV-2 viruses ^a^	40	62.5	16.5	16.5
Any respiratory virus ^b^	64	100	26.4	34.4

^a^ Three cases tested positive for two viruses. ^b^ Six cases tested positive for two viruses.

**Table 2:** Results for SARS-CoV-2 and other respiratory virus by epidemiological and clinical characteristics.

	Total with Valid Result *N* (%)	Any Respiratory Virus *n* (% of Testers)	*p* Value	SARS-CoV-2 *n* (% of Testers)	*p* Value	Other Respiratory Virus *n* (% of Testers)	*p* Value
**Total**	242 (100)	64 (26.4)		27 (11.2)		40 (16.5)	
**Sex**			0.830		0.874		0.773
Male	122 (50.4)	33 (27.0)		14 (11.5)		21 (17.2)	
Female	120 (49.6)	31 (25.8)		13 (10.8)		19 (15.8)	
**Age, years**			0.048		0.184		0.242
<70	42 (17.4)	8 (19.0)		2 (4.8)		6 (14.3)	
70–79	41 (16.9)	8 (19.5)		4 (9.8)		4 (9.8)	
80–89	76 (31.4)	17 (22.4)		7 (9.2)		11 (14.5)	
≥90	83 (34.3)	31 (37.3)		14 (16.9)		19 (22.9)	
**Month**			0.033		0.015		0.055
December	57 (23.6)	16 (28.1)		2 (3.5)		14 (24.6)	
January	28 (11.6)	7 (25.0)		4 (14.3)		3 (10.7)	
February	35 (14.5)	8 (22.9)		7 (20.0)		2 (5.7)	
March	52 (21.5)	7 (13.5)		1 (1.9)		6 (11.5)	
April	37 (15.3)	17 (44.7)		8 (21.6)		10 (26.3)	
May	24 (9.9)	8 (33.3)		4 (16.7)		5 (20.8)	
June	9 (3.7)	1 (11.1)		1 (11.1)		0 (0.0)	
**Respiratory symptoms**			0.467		0.093		0.115
Yes	109 (45.0)	32 (29.4)		9 (8.3)		24 (22.0)	
No	75 (31.0)	16 (21.3)		7 (9.3)		9 (12.0)	
Unknow	58 (24.0)	16 (27.6)		11 (19.0)		7 (12.1)	
**Hospitalized ≤ 30 days before death**			0.114		0.454		0.165
Yes	117 (48.3)	34 (30.7)		13 (12.6)		22 (19.7)	
No	125 (51.7)	30 (24.0)		14 (119.6)		18 (13.0)	
**Pre-mortem diagnosis ≤ 30 days before death**			<0.001		<0.001		0.909
Yes	13 (5.4)	11 (84.6)		9 (69.2)		2 (15.4)	
No	229 (94.6)	53 (23.1)		18 (7.9)		38 (16.6)	
**Influenza vaccination**			0.424		0.415		0.091
Unvaccinated	70 (28.9)	21(30.0)		6 (8.6)		16 (22.9)	
Vaccinated	172 (71.1)	43 (25.0)		21 (12.2)		24 (14.0)	
**COVID-19 vaccination**			0.013		0.523		0.022
Unvaccinated	10 (4.1)	6 (60.0)		1 (10.0)		6 (60.0)	
Vaccinated without booster dose	36 (14.9)	13 (36.1)		6 (16.7)		8 (22.2)	
Vaccinated and booster dose	196 (81.0)	45 (23.0)		20 (10.2)		26 (13.3)	

## Data Availability

The data presented in this study are available on request from the corresponding author.
